# Balance and gait in progressive supranuclear palsy: a narrative review of objective metrics and exercise interventions

**DOI:** 10.3389/fneur.2023.1212185

**Published:** 2023-06-23

**Authors:** Marian L. Dale, Carla Silva-Batista, Filipe Oliveira de Almeida, Fay B. Horak

**Affiliations:** ^1^Balance Disorders Laboratory, Department of Neurology, Oregon Health and Science University, Portland, OR, United States; ^2^Neurology Section, VA Portland Health Care System, Veterans Health Administration, Portland, OR, United States; ^3^Exercise Neuroscience Research Group, University of São Paulo, São Paulo, Brazil

**Keywords:** progressive supranuclear palsy, balance, gait, objective measurements, rehabilitation

## Abstract

**Background:**

The use of objective gait and balance metrics is rapidly expanding for evaluation of atypical parkinsonism, and these measures add to clinical observations. Evidence for rehabilitation interventions to improve objective measures of balance and gait in atypical parkinsonism is needed.

**Aim:**

Our aim is to review, with a narrative approach, current evidence on objective metrics for gait and balance and exercise interventions in progressive supranuclear palsy (PSP).

**Methods:**

Literature searches were conducted in four computerized databases from the earliest record up to April 2023: PubMed, ISI’s Web of Knowledge, Cochrane’s Library, and Embase. Data were extracted for study type (cross-sectional, longitudinal, and rehabilitation interventions), study design (e.g., experimental design and case series), sample characteristics, and gait and balance measurements.

**Results:**

Eighteen gait and balance (16 cross-sectional and 4 longitudinal) and 14 rehabilitation intervention studies were included. Cross-sectional studies showed that people with PSP have impairments in gait initiation and steady-state gait using wearable sensors, and in static and dynamic balance assessed by posturography when compared to Parkinson’s disease (PD) and healthy controls. Two longitudinal studies observed that wearable sensors can serve as objective measures of PSP progression, using relevant variables of change in turn velocity, stride length variability, toe off angle, cadence, and cycle duration. Rehabilitation studies investigated the effect of different interventions (e.g., balance training, body-weight supported treadmill gait, sensorimotor training, and cerebellar transcranial magnetic stimulation) on gait, clinical balance, and static and dynamic balance assessed by posturography measurements. No rehabilitation study in PSP used wearable sensors to evaluate gait and balance impairments. Although clinical balance was assessed in 6 rehabilitation studies, 3 of these studies used a quasi-experimental design, 2 used a case series, only 1 study used an experimental design, and sample sizes were relatively small.

**Conclusion:**

Wearable sensors to quantify balance and gait impairments are emerging as a means of documenting progression of PSP. Robust evidence for improving balance and gait in PSP was not found for rehabilitation studies. Future powered, prospective and robust clinical trials are needed to investigate the effects of rehabilitation interventions on objective gait and balance outcomes in people with PSP.

## Introduction

Progressive supranuclear palsy (PSP) is a relatively rare and rapidly progressive neurodegenerative disease classified among atypical Parkinsonisms (1, 2), but evidence suggests that the clinical spectrum of PSP is larger than originally described. The most frequent form of the disease, PSP-RS (PSP Richardson syndrome), is characterized by vertical supranuclear gaze palsy and backward postural instability with early falls (2), while the second most common form of disease is characterized by a parkinsonian syndrome resembling Parkinson’s disease (PD) especially in the earliest stages (3). The 2017 Movement Disorder Society criteria recognize multiple subtypes of PSP (4), and these subtypes encompass a spectrum of degree of gait and balance deficits. PSP-RS, PSP-P, and PSP-progressive gait freezing (PSP-PGF) display prominent gait and balance abnormalities, while other subtypes and stages of PSP, such as probable PSP frontal presentation (probable PSP-F) and possible PSP speech and language (possible PSP-SL), are characterized primarily by deficits other than gait and balance impairment.

It has long been recognized that particular clinical exam findings and history questions serve as a red flag for gait and balance in atypical parkinsonism disorders, such as PSP and multiple system atrophy (MSA). For example, Nonnekes et al. (5) highlighted the *tandem gait sign* and *bicycle sign* as indicative of atypical parkinsonism versus idiopathic PD (iPD): if a patient has impaired tandem gait or states that early in their disease course that they were no longer able to ride a bicycle, one should be concerned for possible atypical parkinsonism. This reflects the clinical observation of a wider-based gait and earlier balance troubles as reflective of atypical parkinsonism.

The use of objective gait and balance metrics is rapidly expanding for evaluation of atypical parkinsonism, and these measures add to clinical observations. For example, Raccagni et al. (6) used inertial sensors on the feet to compare a group of subjects with PSP and MSA to a group with iPD and found reduced gait speed and stride length in the atypical parkinsonism subjects compared to subjects with iPD.

Although advances in technology of small, body-worn, inertial sensors have objectively quantified balance and gait impairments in the clinic for research trials and clinical practice in people with PD (7–9), this approach has not been explored in PSP. Objective balance and gait metrics may eventually provide useful biomarkers for PSP, clinical efficacy of new treatments, in place of counting falls from diaries or clinical balance rating scales. Objective balance and gait biomarkers also may be helpful in clinical practice to monitor effects of interventions and prognosis. Biomarkers of balance control could be especially useful to monitor PSP progression and fall risk as well as to differentiate PSP subtypes.

In this narrative review, we examine current evidence for objective metrics of gait and balance in people with PSP. We summarize cross-sectional studies examining gait initiation, steady state gait, and balance in PSP, as well as studies that use gait and balance data mining approaches for classification of PSP, and studies examining radiological correlations with gait and balance metrics in PSP. We then discuss the emerging use of objective gait and balance measures for longitudinal monitoring in PSP and objective gait and balance measures as endpoints for rehabilitation intervention trials in PSP.

## Methods

Literature searches were conducted in the following four computerized databases from the earliest record up to April 2023: PubMed, ISI’s Web of Knowledge, Cochrane’s Library, and Embase. Inclusion criteria were: any study design (cross-sectional, longitudinal, and rehabilitation interventions) published in peer-reviewed journal, published in English, available in full text, with or without rehabilitation interventions [e.g., physical exercise, virtual reality, and repetitive transcranial magnetic stimulation (rTMS)], population with diagnosis of PSP, mixed PSP subtypes, gait and/or balance assessment. Exclusion criteria were: no gait and/or balance assessment and invasive brain stimulation.

**Figure 1:**
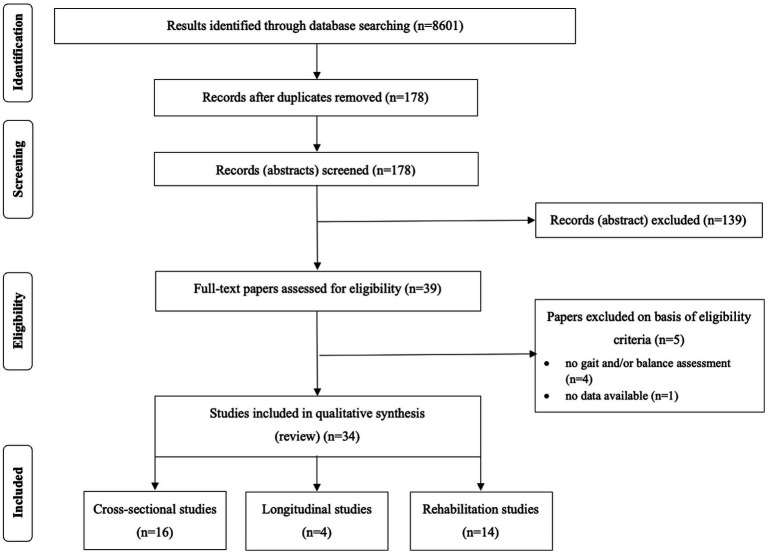
PRISMA flowchart for inclusion into the review.

The search was limited to English language. All the identified and retrieved electronic search titles, selected abstracts, and full-text articles were independently evaluated by two of the authors (FOA and CSB) to assess their eligibility. In case of disagreements, a consensus was adopted or, if necessary, a third reviewer evaluated the article (MD). The search process is depicted in Figure 1.

## Results

### Gait and balance as a diagnostic tool: cross-sectional studies

Sixteen cross-sectional studies were included in this review (Table 1). These studies compared gait initiation, steady state gait, and balance between people with and without PSP. In addition, some studies used radiological correlations with gait and balance measures in PSP and mixed PSP phenotypes. We have separated the following discussion of cross-sectional studies according the type of gait and balance assessment.

**Table 1:** Summary characteristics of the cross-sectional included studies.

Study and Country	Participants	Subtypes of PSP and criteria for PSP	Cognitive and Mood status	Assessment	Walking aids (YES/NO) and type	Measurement Tool	Clinical motor outcomes/other outcomes	Objective gait outcomes	Objective balance outcomes	Correlational analysis	Author’s conclusion
Ali et al. (20) USA	PSP: *n=*16, age (70.4±7.1 years), disease duration (4.4±2.8 years), men (*n=*10), women (*n=*6) HC: *n=*25, age (72.7±6.6 years), women (*n=*25)	PSPRS UPDRS-III Richardson’s syndrome (*n=*10), Cortico-basal syndrome (*n=* 3), Parkinsonism predominant (*n=*2), Speech and language disorder (*n=*2), Frontal predominant (*n=*1)	Not Reported	Patients were not on any dopaminergic medications.	Not reported	Force plate, 3D motion system	UPDRS-III scores were average 50 (range: 20, 85) for PSP, while PSPRS scores average was 39 (range:24, 58).	PSP patients walked with a slower velocity, lower cadence, shorter stride, and step lengths, and reduced single support times compared to healthy older adults. Total sagittal plane ROM in the hip, knee and ankle showed significant decreased ROM (*p<*0.05) when comparing patients with PSP to healthy adults	PSP exhibited significantly larger amplitudes of COP displacement (7.0±3.9) compared to the healthy individuals (3.4±2.2) for the eyes open task (*p<*0.01). PSP patients exhibited less displacement in the ML (2.2±1.1) but significantly increased displacement in the AP (7.2±4.0) direction compared to the healthy individuals (5.1±1.6) in the eyes closed task (*p<*0.04).	There were significant correlations between PSPRS and UPDRS with gait velocity, (rs=0.597, *p=*0.015; rs=0.756, *p=*0.001), total support (rs=0.591, *p=*0.016; rs=0.546, *p=*0.029), single support (rs=0.557, *p=*0.025; rs=0.500, *p=*0.049), and step length (rs=0.561, *p=*0.024; rs=0.764, *p=*0.001). Significant correlations were also found for UPDRS only and initial double support (rs=0.582, *p=*0.018) and hip ROM (rs=0.728, *p=*0.003).	Patients with PSP have increased anteroposterior sway, slower gait velocity, wider stance, and lower cadence. The gait stability ratio and Romberg ratio was high consistent with postural imbalance and increased reliance on vision for stability, experienced by PSP patients. Motion analysis metrics correlated with clinical scales reflecting that they are a marker of disease severity.
Amano et al. (10) USA	PSP: *n=* 12, age (66 ± 8.0 years), disease duration (6.5 ± 4.9 years), men (*n=*5), women (*n=*7) PD: *n=*12, age (64 ± 7 years), disease duration (7.8 ± 7.1 years), men (*n=*5), women (*n=*7) HC: *n=*12, age (67 ± 7 years), men (*n=*5), women (*n=*7)	NINDS-SPSP UPDRS Subtypes not reported	Not reported.	ON-assessment	Not reported	3D motion system	UPDRS-III (PSP: 49.6±10.4; PD: 23.5±8.5). UPDRS PIGD (PSP:6.33±2.46; PD:3.33±2.42)	The PSP group exhibited significantly reduced cadence, gait velocity, step length, and step duration compared to the other two groups.	COP displacement AP (PSP 0.71±1.55, PD –1.14±0.71, HC –2,61±1.56) COP displacement ML (PSP 2.46±3.62, PD –0.81±1.03, HC –1.85±1.28) The maximum distance between COP and COM significantly differed among the groups.	Not reported.	The study identified significant differences in specific biomechanical characteristics during gait initiation and gait between PSP and PD. Abnormally shorter and slower step during GI in PSP was observed and may result from the inability to execute APAs. The compensatory GI strategy, characterized by diminished posterior COP shift and weight shift toward the stance limb, is therefore very distinct from PD and paradoxically induces lateral postural instability. PSP gait, which prioritizes stability over mobility, may be compensatory and could be the consequence of lateral instability and fear of falling.
Amboni et al. (14) Italy	PSP Total: *n=* 21, age (67.8 ± 7.4 years), disease duration (2.5 ± 1.1 years), men (*n=*11), women (*n=*10) PD Total: *n=*83, age (63.2 ± 8.5 years), disease duration (3.4 ± 3.2 years), men (*n=*55), women (*n=*28) Early PSP: *n=* 12, age (63.5 ± 5.9 years), disease duration (1.7 ± 0.4 year), men (*n=*7), women (*n=*5) De novo PD: *n=*27, age (63.3 ± 8.7 years), disease duration (< 1 year), men (*n=*17), women (*n=*10)	PSPRS NDS-UPDRS-III Richardson’s syndrome (*n=*11), Parkinsonism predominant (*n=*5), Freezing of gait predominant (*n=*4)	MMSE: PSP Total (25.1 ± 3.0), PD Total (26.8 ± 2.3), Early PSP (25.5 ± 2.7), De novo PD (26.9 ± 2.2)	*ON-assessment*	No	SMART DX system (3D motion system + force plate)	MDS-UPDRS-III (PD Total): 19.46±8.99; MDS-UPDRS-III (De novo PD): 12.85±6.17; PSP-RS-V (PSP total): 5.81±2.73; PSP-RS-VI (PSP total): 7.28±4.55; PSP-RS-V (Early PSP): 5,.3±2.87 PSP-RS-VI (Early PSP): 6.58±3.73	Compared to PD, PSP patients exhibited reduced velocity and cadence, shortened step and cycle lengths, increased cycle duration mainly due to longer double support stance phase duration, and increased swing duration variability during single task. During dual task, PSP patients exhibited the same gait features as those displayed during the single task, except for swing duration and step length variability. Compared to newly diagnosed PD patients, early PSP patients exhibited reduced velocity and cadence, shortened step and cycle length, and increased cycle duration; these patients tended to rely on a longer double support stance phase during single task. During dual task, early PSP patients exhibited a gait pattern similar to that during the single task except for swing duration and swing duration variability.	Not reported.	Not reported.	The study demonstrates that quantitative gait evaluation clearly distinguishes PSP patients from PD patients since the earliest stages of disease. These findings indicate that gait analysis could be a candidate as a reliable biomarker in both clinical and research setting. In addition, results may offer speculative clues for conceiving early disease-specific rehabilitation strategies.
Dale et al. (19) USA	PSP: *n=* 12, age (70 ± 6.3 years), disease duration (2.6 ± 1.5 years), men (*n=*6), women (*n=*6) PD: *n=*12, age (67.8 ± 7.3 years), disease duration (8.1 ± 5.6 years), men (*n=*6), women (*n=*6) HC: *n=*12, age (not reported), men (not reported), women (not reported)	NINDS-SPSP UPDRS Subtypes not reported	Not reported	*OFF-*assessment	Not reported	NeuroCom Balance Master Clinical Research System	UPDRS-III (PD): 33.7 ± 7.5; UPDRS-III (PSP): 34.4 ± 9.1; PSP-RS: 26.9 ± 11.9	Not reported	PSP displaced their CoP significantly less than PD subjects (p≤0.006) and slightly less than healthy subjects during the forward translation of the platform. The CoP of subjects with PSP remained more posterior after the platform shifted back to the initial position compared to subjects with PD (p≤0.01), while only a slight difference was found compared to healthy subjects. When the body was displaced backward by toes-up platform rotation, PSP exerted a significantly larger destabilizing plantar-flexion torque (as evidenced by forward CoP displacement) than subjects with PD (p≤0.008), and only slightly larger compared to healthy subjects.	Not reported	The study demonstrates inappropriate adaptive postural motor control with excessive forward CoP displacement in response to toes-up surface tilts in PSP.
De Vos et al. (22) United Kingdom	PSP: *n=* 21, age (71 years), disease duration (2 years), men (*n=*12), women (*n=*9) PD: *n=*20, age (66.4 years), disease duration (11.4 years), men (*n=*11), women (*n=*9) Healthy Control: *n=*39, age (67.1 years), men (19), women (20)	UPDRS-III Richardson’s syndrome (*n=*4), Parkinsonism predominant (*n=*17)	MoCA: PSP (mean 22), PD (mean 26.6), HC (mean 28.5). MMSE: PSP (mean 25.8), PD (mean 26.6), HC (mean 27.6).	*ON-*assessment	Not reported	IMUs	UPDRS-III (PSP: 44.6; PD: 27.9)	Gait cadence distinguished PSP from PD and HC showing a high specificity (90 %) when using 6 sensors (Mobility Lab™, APDM) over the lumbar spine, sternum, left and right wrists, and left and right feet.	Mean postural sway velocity in the coronal plane during the sway test, mean time taken to sit from standing during the timed up-and-go (TUG) task, mean time taken to turn during the gait task, mean time taken to turn during the TUG task, standard deviation of time taken to turn during the gait task distinguished PSP from PD and HC showing a high specificity (90 %) when using 6 sensors (Mobility Lab™, APDM) over the lumbar spine, sternum, left and right wrists, and left and right feet.	Not reported	A wearable inertial measurement unit array and machine learning methods can accurately differentiate PSP from PD and from control.
Hatanaka et al. (12) Japan	PSP: *n=* 20, age (71,8 ±5.9 years), disease duration (3.4±1.8 years), men (*n=*14), women (*n=*6) PD: *n=*124, age (68.4 ± 11.2 years), disease duration (6.7±7.4 years), men (*n=*64), women (*n=*60) HC: *n=*24, age (73.7 ± 3.8 years), men (5), women (19)	NINDS-SPSP Subtypes not reported	Not Reported	*ON-*assessment	No	Portable triaxial accelerometer rhythmogram device	Not reported	Compared with the accelerogram of HC, both PSP and PD patients showed a smaller amplitude (acceleration) and increased shuffle frequency over a certain period, indicating a reduced acceleration and shorter step time. Compared with HC (1.10 ± 0.22 m/s), velocity was reduced in PSP patients (0.83 ± 0.23 m/s, p < 0.01 vs. control) and in all PD patients (0.89 ± 0.24 m/s, p < 0.01). The cadence of the PSP patients (100.5 ± 11.5 steps/min, p < 0.01 vs. control) was significantly lower than HC (115.9 ± 11.2 steps/min) and of PD patients (109.3 ± 15.9 steps/min, # p < 0.05 vs. PSP). The vertical displacement of PSP patients (2.3 ± 1.1 cm) was significantly lower than HC (5.6 ± 1.7 cm, *p<* 0.01 vs. PSP), all PD patients (4.4 ± 2.2 cm, *p<* 0.01 vs. PSP).	Not reported	There was a close relationship between cadence and acceleration for all groups. The relationships were positive and linear with R^2^ values > 0.4 (controls, R^2^ = 0.54; PSP patients, R^2^ = 0.56; PD patients, R^2^ = 0.48).	Lower vertical displacement could be a feature of gait disturbance in PSP patients, and which could be used to better discriminate PSP from PD patients.
Liao et al. (17) USA/ Germany	PSP-tVOR: *n=*9, age (median 68 years, range 61-75), men (*n=*5), women (*n=*4) PSP-VEMPs: *n=*10, age (median 68 years, range 60-76), men (*n=*7), women (*n=*3) HC-tVOR: *n=*9, age (median 67 years, range 60-72), men (*n=*6), women (*n=*3) HC-VEMPs: *n=*30, age (median 67 years, range 56-80), men (*n=*19), women (*n=*11)	NINDS-SPSP Subtypes not reported	Not reported	Not reported	Not reported	Magnetic search coil + Infrared reflection system	Not reported	Not reported	*Vestibulo-ocular reflex:* Patients with PSP tend to show smaller values of aVOR RR than control subjects, but with some overlap of data. For tVOR, patients with PSP’s RR were smaller than controls during far viewing, but with overlap of data. However, during near-viewing conditions, PSP RR values for tVOR were significantly smaller than controls, with no overlap of data. The range of tVOR RR of our control subjects was similar to those previously reported during rapid oscillatory head translations, but responses of patients with PSP during near viewing were, on average, only 12% of controls. *Vestibulo-spinal reflex:* The median P1-N1 amplitude of all 60 ears of the HC was 149 μV (range: 11.6 to 466); that of all 20 ears of the patients with PSP 54.3 μV (range: 16.8 to 214).	Not reported	The study results indicate that abnormal otolith-mediated reflexes may be at least partly responsible for frequent falls in progressive supranuclear palsy.
Ondo et al. (18) USA	PSP: *n=* 20, age (68 ±5.4 years), disease duration (3.5±1.5 years), men (*n=*8), women (*n=*12) PD: *n=*20, age (65.4 ± 5.3 years), disease duration (4.4±1.5 years), men (*n=*13), women (*n=*7) HC: *n=*20, age (69 ± 3 years), men (8), women (12)	Clinical criteria Subtypes not reported	Not reported	*OFF-*assessment	Not reported	Computerized posturography	PSP group was significantly worse than both other groups (POAG, z = −5.13 [*P<*.001]; POAB, z = −5.02 [*P<*.001]). The FR measures showed significant differences among the groups (F2 = 48.2; *P<*.001, univariate ANOVA). PSP group scores were significantly lower than those of both other groups (*P<*.001), and that PD group scores were significantly lower than those of controls (*P=* .003).	Not reported.	The total stability score (SOT) showed that PSP group total scores were worse than those of both other groups (*P<*.001). CT sway was greater in the PSP than in the PD or control groups (*P=*.02); and total LOS measures of path time to target (*P<*.001) and path sway from a straight line to target (*P<*.001) were significantly prolonged in the PSP compared with the PD and control groups. The PSP group tended to have better performance with lateral movement and worse with anterior/posterior movements	Total LOS correlated with total POA scores (Pearson correlation, 0.67).	Results demonstrate significant abnormalities of postural control in patients with PSP that were markedly worse than those seen in a PD group matched for age and disease duration, and in age-matched healthy controls. Although clinical assessments also showed significant differences among the groups, CP more accurately discriminated early PSP from early PD.
Palmisano et al. (24) Italy	PSP: *n=* 20, age (66.6 ±4.7 years), disease duration (5.3±3.1 years), men (*n=*6), women (*n=*14) HC: *n=*25, age (65.1 ± 3.4 years), men (9), women (14)	PSPRS All had Richardson’s Syndrome	Not reported	*OFF-*assessment	Not reported	Force plate, 3D motion system	PSPRS Total (33 ± 9,7); PSPRS limb motor (5.6± 1.9); PSPRS gait and midline (9.3 ± 2.7) *Brain metabolic measures:* there are six hypometabolic brain regions in the PSP group: the right dorsolateral prefrontal cortex, the left supplementary motor area, the middle cingulate cortex, the left caudate Nucleus, the medial thalamus and the Midbrain.	Not reported.	CoM movement was significantly impaired in the PSP group as revealed by lower values of the velocity and acceleration of the CoM at the unloading phase end, as well as the velocity and position of the CoM with respect to the CoP at the stance foot toe-off. Patients with PSP also showed a significantly reduced first step length, average and maximum velocity compared to HC.	In healthy controls, the velocity, acceleration and position of the CoM with respect to the CoP at IMB end were influenced by AM and BoS parameters. In the same cohort, during the unloading phase the CoP displacement and the average and maximum velocity of the CoP in the ML direction were influenced by the BoS. In the PSP group, no correlations of GI parameters with BoS measurements were found. The study also showed a significant correlation between the PSPRS subscores related to motor impairment (i.e., Limb motor, and Gait and midline) and the kinematic measurements of all GI phases. The caudate nucleus, together with the middle cingulate cortex, correlated with the velocity of the CoM at the end of the unload phase and, together with the thalamus, with the distance between CoP and CoM at stance foot toe-off.	The results of the study provide evidence to support the hypothesis that dysfunctional postural control at GI in PSP patients involves poor APA programming and execution. Multiple brain regions of the supraspinal locomotor network specifically contributes in a principled, controlled manner to an efficient GI.
Pasha et al. (23) India	PSP: *n=* 29, age (60.8 ± 8.2 years), disease duration (2.2±1.2 years), men (*n=*21), women (*n=*8) HC: *n=*30, age (59.8 ± 7.6 years), men (17), women (13)	NINDS-SPSP Richardson’s syndrome (17); Parkinsonism predominant (12)	MMSE: PSP (24.8±5.04); HC (29.7 ± 1.0)	ON and OFF-assessments	Not reported	Dynamic posturograph*y*	PSPRS, TPG, and TPT scores were found to be statistically significant among the subtypes of PSP (*P=*0.045; *P=*0.031; and *P=*0.037) while UPDRS-III was not significant (*P=*0.7). The PSP-R subtype performed poorly in comparison to the PSP-P subtype on these scales. *MRI measures:* The PSP-R subtype, compared to the PSP-P subtype, had more often radiological signs such as HBS (P < 0.001), MGS (P <0.008), and GCA (P <0.001).		The mean values of balance indices were almost similar between the subtypes of PSP as compared to controls. The most significant of all the parameters in DP was LOS (P < 0.001) and the PSP-R subtype had lower scores.	There was a significant correlation of PSPRS with BBS (r = −0.642, P < 0.001), TPT (r = −0.516, *P=*0.004), TPG (r = −0.449, *P=*0.013), and TPB (r = −0.505, *P=*0.004). LOS-BW-LT had significant positive correlation with BBS (r = 0.381, *P=*0.038), TPB (r = 0.417, *P=*0.022), and TPT scores (r = 0.362, *P=*0.049). There was a significant correlation of the midbrain axial AP diameter (r = 0.4; *P=*0.03) and the ratio of midbrain to pons with BBS (r = 0.4; *P=*0.02), indicating that these are worse in patients with midbrain atrophy. In DP, API correlated negatively with the midbrain axial AP diameter (r = −0.5; *P=*0.01) and midbrain area (−0.39; *P=*0.03). The LOS-BW correlated positively with the area of midbrain (r = 0.49; *P=*0.001) and the midbrain to pons ratio (r = 0.57; r = 0.001). In addition, LOS-BW-LT correlated with the midbrain to pons ratio (r = 0.37; *P=*0.43)	The study shows that the measurements of balance severity in the PSP-P group correlate with the predominant pathology of the midbrain (midbrain atrophy); while in PSP-R subtype, the balance abnormalities could be a result of pathology in different or overlapping areas.
Picillo et al. (16) Italy	PSP-RS: *n=* 10, age (69.9 ±7.6 years), disease duration (2.5 ± 1.17 years), men (*n=*5), women (*n=*5) PSP (other subtypes): *n=*9, age (66.5 ± 5.9 years), disease duration (2.33 ± 1 years), men (*n=*6), women (*n=*3)	MDS-UPDRS-III PSPRS Richardson’s syndrome (*n=*10), Parkinsonism predominant (*n=*5), Freezing of Gait predominant (*n=*4)	MoCA: PSP-R (15.3 ± 5.7), PSP-other (19.5 ± 4.2).	Not Reported	No	SMART DX system (3D motion system + force plate)	PSPRS (PSP-R: 34.9 ± 13.8; PSP-other: 28.33 ± 9.38)	PSP-R showed worse gait parameters than did other subtypes of PSP during single task. In detail, PSP-RS exhibited reduced cadence and increased cycle duration (*p=*0.018), mainly due to longer stance duration (*p=*0.034). For the dual task analysis, PSP-RS continued to roughly show the same gait features displayed during the single task. In addition, PSP-RS showed increased stance phase and reduced swing phase (*p=*0.031). There was a trend for significance for greater variability in step length (*p=*0.069) and lower velocity (*p=* 0.098) in PSP-RS.	Not reported	In patients with PSP-RS, constructional apraxia and right ideomotor apraxia presented an inverse relationship with cycle and swing duration and a direct correlation with cadence (*p<*0.05). TMT parts A and B showed a direct correlation with swing duration and cycle duration, respectively (*p<*0.05). No significant correlations were shown for other subtypes of PSP.	PSP-RS presents greater gait dynamic instability since the earliest stages of disease compared with other subtypes of PSP. In addition, these findings indicate that gait quantitative evaluation can help to distinguish PSP-RS from other subtypes of PSP.
Raccagni et al. (6) Austria	PSP: *n=* 12, age (67.4 ±8.7 years), disease duration (5.0±3.6 years), men (*n=*9), women (*n=*3) PD: *n=*25, age (66.6 ± 7.9 years), disease duration (7.5±4.5 years), men (*n=*13), women (*n=*12) HC: *n=*25, age (63.7 ± 9.7 years), men (13), women (12)	MDS-UPDRS-III PSPRS Subtypes not reported	Not reported	*ON-*assessment	Not reported	Wearable sensor-based gait analysis system	MDS-UPDRS-III (PSP: 41.7*±*15.5; PD: 31.7±9.3)	Gait speed was significantly reduced in PD patients (1.20 ± 0.23m/s) compared to controls 1.38 ± 0.20 m/s; *p=*.011) and even more impaired in APD patients (0.98 ± 0.18 m/s). 1.38 ± 0.20 m/s; *p=*.011) and even more impaired in APD patients (0.98 ± 0.18 m/s). Results showed significant difference for stride length in controls (1.47 ± 0.15 m), PD (1.27 ± 0.22 m) and APD (1.11 ± 0.18m). Maximum toe clearance was significantly reduced in PD (7.8 ± 2.6 cm; *p=*.001) and APD patients (6.9 ± 2.8 cm; *p=*.000) compared to controls (10.8 ± 3.3 cm)	Not reported	Stride length correlated with PSP-RS scores in the PSP patients (r=0.59, *p=*.021). There was a significant correlation between maximum toe clearance and MDS-UPDRS-3 (r =−.444, *p=*.026) in APD.	The significant difference of objective gait parameters among patient groups suggests that sensor-based technology may support and complement the clinical assessment provided by validated rating scales.
Ricciardi et al. (21) Italy	PSP: *n=* 7, age (70.5 ±8.9 years), disease duration (2.6±0.5 years), men (*n=*3), women (*n=*4) De novo PD: *n=*15, age (63.3 ± 6.7 years), disease duration (0), men (*n=*11), women (*n=*4) Stable PD: *n=*24, age (61.9 ± 6.7 years), disease duration (5.9±2.5), men (17), women (7)	MDS-UPDRS-III PSPRS Richardson’s syndrome (*n=*4); Parkinsonism predominant (*n=*3)	Not reported	Not reported	No	SMART DX system (3D motion system + force plate)	MDS-UPDRS-III (De novo PD: 14.3±7.5; Stable PD: 21.3±6.3), PSPRS (16.8±3.3)	The PSP group showed the highest sensitivity and specificity among all patients, both overcame the threshold of 90% and, particularly, the specificity went beyond 95%. De Novo PD’s sensitivity and specificity scores were remarkable as well as the previous group, getting close to the value of 90%. The Stable PD group achieved the lowest sensitivity (between 65% and 70%) but high specificity; this metric, that represents the capacity to classify correctly the examined group but not the others, overcame the value of 90%.	Not reported	Not reported	The study’s methodology allowed a good overall accuracy and re- markable sensitivities in the classification of PSP and De Novo PD patients. This indicates that the present approach could provide the clinician with a reliable, low-cost, non-invasive tool to distinguish early PSP from PD, in the first phases of the diseases’ courses when the diagnosis of atypical forms of Parkinsonism is challenging.
Selge et al. (13) Germany	PSP: *n=* 38, age (69 ±6.3 years), disease duration (3.5 ± 2.2), men (*n=*20), women (*n=*18) iNPH: *n=*27, age (63.3 ± 6.7 years), disease duration (1.9 ± 1.6), men (*n=*21), women (*n=*6) HC: *n=*38, age (68.9 ± 7.6 years), men (20), women (18)	NINDS-SPSP Subtypes not reported	MMSE (PSP: 27.3 ± 3.1; iNPH; 23.4 ± 3.3) and FAB (PSP: 13.9 ± 2.3)	Not reported	No	GAITRite (6.7-m-long pressure-sensitive carpet system)	PSPRS (31.1±8.9)	Compared to HC, both patients with PSP and those with iNPH had a significantly inferior gait performance. Compared with patients with PSP, the gait of patients with iNPH was characterized by a lower velocity (*p=*0.001) and shorter stride length (p < 0.001). The main differences were a more broad-based gait in iNPH (p < 0.001) and a higher CV of stride time in PSP (*p=*0.009). Cognitive dual task led to a significant impairment of gait in all 3 groups. Patients with PSP were significantly more sensitive to dual-task perturbation than patients with iNPH. Especially gait velocity was clearly more reduced in patients with PSP, while the reduction in patients with iNPH was comparable to that in HC. Motor dual task led to a significant decrease of gait velocity and stride length in patients with PSP, but to a lesser extent than cognitive dual task.	Not reported	In patients with PSP, the CV of stride length increased at the time when the CV of step width decreased (r = −0.338). This correlation was not seen in patients with iNPH or HC.	Compared with patients with PSP, the gait of patients with iNPH was slower and broader based; gait variability was higher in patients with PSP; and patients with PSP were more sensitive to dual-task perturbation. Under motor dual task, patients with iNPH tended to even improve.
Sintini et al. (25) USA	PSP: *n=* 19, age (71 ±7 years), disease duration (4.7 ± 2.6), men (*n=*11), women (*n=*8)	MDS-UPDRS-III PSPRS Richardson’s syndrome (*n=*13), Parkinsonism predominant (*n=*3), Cortico-basal syndrome (*n=* 1), Speech and language disorder (*n=*2)	Not reported	Not reported	No	Force plate, 3D motion system	PSPRS (39 ± 10); MDS-UPDRS-III (49 ± 17) *Neuroimaging measures:* MRI atrophy, white matter tracts degeneration and flortaucipir-PET uptake were measured. Typically, DTI-FA and MRI volumes are reduced in PSP relative to healthy controls, while DTI-MD and flortaucipir SUVR are increased.	Stride length, cadence, velocity and step width were measured. Compared to HC, velocity, cadence and stride length are typically lower in PSP patients, while step width and stride length coefficient of variation are typically higher.	Gait stability ratio, total support, initial double support, postural imbalance and dynamic stability were measured. Compared to HC, velocity, cadence, stride length and dynamic stability are typically lower in PSP patients, while step width, gait stability ratio, total support, initial double support, postural imbalance, and stride length coefficient of variation are typically higher.	PSP rating scale and MDS-UPDRS III scores strongly correlated to velocity, stride length, gait stability ratio and dynamic stability. The gait midline sub-scale score of the PSP rating scale strongly correlated to velocity, dynamic stability, and stride length CV. Velocity was negatively correlated to DTI-MD in the cerebellar peduncle and positively correlated to volume in the supplementary motor area, superior frontal, and lateral parietal cortex. Velocity was positively correlated to subcortical flortaucipir-PET uptake (subthalamic nucleus, pallidum, caudate, red nucleus). Cadence was positively associated with DTI-MD in various tracts, especially the sagittal stratum and the cingulum (hippocampus). Stride length was strongly associated with DTI-MD in the body and splenium of the corpus callosum and with volume in the precentral, superior, and medial frontal, and parietal cortex. Step width was strongly related to DTI-MD in the superior cerebellar peduncle and flortaucipir-PET uptake in the dentate nucleus, cerebellum, and pons. Total support time was higher (i.e., more impaired gait) in patients with lower DTI-FA in many tracts, particularly the posterior thalamic radiation, sagittal stratum, external capsule and splenium of the corpus callosum. Greater postural imbalance with eyes open correlated to reduced metabolism in the cerebellar crus and lateral parietal cortex and greater postural imbalance with eyes closed correlated to reduced metabolism in the dentate nucleus. Lower dynamic stability strongly correlated with lower volume in the lateral parietal cortex.	The study showed that gait and postural impairments in PSP are associated with imaging abnormalities on different sets of regions and tracts that belong to the PSP system of neurodegeneration and the supraspinal locomotor network. The results suggest that gait and balance impairments might be driven by different mechanisms in PSP.
Takamatsu et al. (15) Japan	PSP: *n=* 27, age (73.4 ± 5.3 years), disease duration (5.0±4.4 years), men (*n=*19), women (*n=*8) PD: *n=*25, age (74.2 ± 5.3 years), disease duration (4.7±3.4 years), men (*n=*14), women (*n=*11) HC: *n=*25, age (73.1 ± 5.3 years), men (11), women (14)	UPDRS-III PSPRS-V and VI Richardson’s syndrome (*n=*19), Progressive gait freezing (*n=*5) Parkinsonism predominant (*n=*3)	Not reported	Not reported	Not reported	WalkWay MW-1000 (2.4-m-long pressure-sensitive carpet system)	PSPRS-V mean (3); PSPRS-VI mean (9); UPDRS-III mean (25)	Walking speed (PSP:75.1± 19.1, HC: 119±21, P < 0.001), CV of cadence (PSP: 5.3 ± 3.9, HC: 2.6±2.5, *P=*0.001), step length (PSP: 42.2 ± 8.9, HC: 62.2 ± 7.1, P < 0.001), step width (PSP:10.6 ± 3.5, HC:7.7 ± 3.4, *P=*0.007), foot angle (PSP: 9.6 ± 6.9, HC: 5,8 ± 4.8, *P=*0.010), time of stance phase (PSP: 0.73±0,12, HC: 0.63± 0,08, *P=*0.031), and double supporting phase (PSP: 0.15±0,04, HC: 0.11±0,02, P < 0.001) showed significant differences between PSP and HC. CV of cadence (PSP: 5.3 ± 3.9, PD: 2.8±2.6, *P=*0.015) and foot angle (PSP: 9.6 ± 6.9, PD: 5.1±4.7, *P=*0.016) showed significant differences between PSP and PD.	Not reported	Not reported	Results suggests that the gait of patients with PSP was unstable with parkinsonism and wide-based, which might be similar to combining features of PD and cerebellar disorders.

A*P=*anteroposterior; APAs = anticipatory postural adjustments; aVOR = angular vestibulo-ocular reflex; BBS = Berg Balance scale; BOS = base of support; CO*P=*center of pressure; COM = center of mass; C*P=*Computerized posturography; CV = coefficient of variation; D*P=*dynamic posturography; DTI-FA = Diffusion tensor imaging—fractional anisotropy; DTI-MD = Diffusion tensor imaging—mean diffusivity; GI = gait initiation; HC = Healthy Control; iNPH = idiopathic normal pressure hydrocephalus; LOS = limits of stability; MDS-UPDRS-III = Movement Disorders Society Unified Parkinson’s Disease Rating Scale part III motor subscale score; ML = mediolateral; MMSE = Mini Mental state examination. MoCA = Montreal Cognitive Assessment; MRI = magnetic resonance imaging; NINDS-SPS*P=*National Institute of Neurological Disorders and Society for Progressive Supranuclear Palsy criteria for PSP; PD = Parkinson’s disease; POA = performance-oriented assessments; POAB = performance-oriented assessment for balance; POAG = performance-oriented assessment for gait; PS*P=*Progressive Supranuclear Palsy; PSPRS = PSP rating scale; PSPRS-V = PSP rating scale-limb motor subscale; PSPRS-VI = PSP rating scale—gait and midline subscale; RR = responsivity ratio; SOT: sensory organization testing; SUVR = standard uptake value ratios; TPB, TPG and TPT = Tinetti performance-oriented mobility assessment (POMA) balance, gait and total; tVOR = translational vestibular-ocular reflex; UPDRS-III = Unified Parkinson’s Disease Rating Scale part III motor subscale score.

#### Gait initiation in PSP

In an elegant 2015 study Amano and colleagues examined the mechanics of gait initiation in PSP using a combination of force platforms embedded in a walkway and a 3D motion capture system (11). Twelve subjects with PSP-Richardson syndrome (PSP-RS), 12 subjects with PD, and 12 age- and gender-matched healthy controls (HC) performed 5 gait initiation trials at a self-selected speed, and their anticipatory postural adjustments (APAs) were examined in detail. Whereas subjects with Parkinson’s disease and HCs displayed the normal APA with an initial backward and lateral center of pressure shift to initiate gait, subjects with PSP could not tolerate the initial destabilization of the APA imbalance phase. In other words, subjects with PSP displayed an inefficient gait initiation strategy because they were unable to initially shift their center of pressure to generate momentum for forward movement, but rather moved their swing foot forward more robotically without the normal, anticipatory weight shift that moves the center of body mass forward and over the stance leg.

The authors proposed that gait initiation in PSP prioritizes stability over mobility, and suggested possible strategies for rehabilitation including focusing on medio-lateral balance to overcome the minimal lateral weight shift and staggering the initial swing foot posteriorly to try to promote the physiological weight shift of a normal APA. Limitations of this study include a lack of accounting for baseline anthropometric measurements and width of the initial base of support, and the fact that all subjects were evaluated *on* levodopa, likely preferentially improving APAs in the iPD group (25). The study was also conducted prior to the 2017 Movement Disorder PSP Criteria (4), and thus only included subjects with the Richardson syndrome variant of PSP (PSP-RS).

#### Steady state gait in PSP-RS

In the same Amano study discussed above, PSP, iPD, and HC subjects also performed 10 steady state gait trials at a self-selected speed, and gait analysis revealed a slower, and more variable, gait in PSP-RS compared to iPD (11). Hatanaka et al. also compared steady state gait in 20 PSP-Richardson syndrome, 124 PD, and 24 HC subjects, using triaxial accelerometers for 10-meter, self-selected straight walking (15). Their study replicated the finding of slower gait in PSP, showing an overall hypokinetic gait pattern with decreased velocity, step length, cadence, and mean acceleration in PSP. They additionally found that the subjects with PSP demonstrated an especially small vertical displacement but larger vertical acceleration than PD patients when comparing subjects with the same cadence.

Selge et al. applied straight walking on a gait mat, with and without cognitive and motor dual tasks, to differentiate PSP-RS from normal pressure hydrocephalus (NPH) (20). Clinically, gait in NPH is considered to be even wider-based and slower than in PSP with an additional “magnetic” quality, but in certain cases the gait patterns of the two diseases approximate each other and contribute to a differential diagnosis that includes both NPH and PSP. In the Selge study, 27 subjects with idiopathic NPH and 38 subjects with PSP performed straight walking at their preferred speed, at a slow speed, and at their maximum speed, as well as dual-task walking at their preferred speed with the serial 7 s cognitive task or while carrying a tray as a motor dual task. Importantly, the PSP and NPH subjects were initially matched on a clinical, functional gait assessment scale. The authors found that gait was slower and more broad-based in NPH, and gait in PSP was more variable and more sensitive to dual-task conditions. They interpreted the increased sensitivity to dual- task conditions in PSP to increased cortical attention for walking. A limitation of this (and many other dual-task studies) was that prioritization of the dual-task was not assessed, so it was not known to what degree the subjects were focused on walking versus on the cognitive task during the assessments.

#### Steady state gait in mixed PSP phenotypes

After establishment of the 2017 Movement Disorder Society Criteria for PSP (4), several groups examined steady state gait in multiple phenotypic variants of PSP. Amboni et al. (12) included variants of PSP to compare with iPD specifically in early diagnostic stages. The iPD subjects were enrolled less than a year from symptom onset and had confirmed positive DAT scans. The subjects with PSP met MDS PSP diagnostic criteria and included 11 with PSP-RS, 5 with PSP-parkinsonism (PSP-P), and 4 with PSP-progressive gait freezing (PSP-PGF). Objective gait analysis revealed a longer stance phase in all PSP variants compared to iPD.

While some groups have compared the gait characteristics of PSP-RS, PSP-P, and PSP-PGF to each other and to iPD, other groups have pooled the variant subtypes to compare as a group to the traditional PSP-RS type, leading to slightly different conclusions. Takamatsu et al. compared gait mat measurements in 27 patients with PSP (including PSP-RS, PSP-PGF, and PSP-P variants) to patients with iPD and to healthy controls (24). They found an overall longer gait cycle time and a larger step width in PSP compared to iPD and HC. They also found a trend toward a faster walking speed in PSP-PGF compared to PSP-RS, and a trend toward a slower walking speed in PSP-P compared to PSP-RS. Low subgroup numbers did not allow for full statistical analysis. Picillo et al. performed a gait analysis in 19 patients with PSP in single and dual tasks (21) and compared the PSP-RS group to a pooled variant group of PSP-P and PSP-PGF (vPSP). Ten of the 19 PSP subjects had the PSP-RS subtype, 5 had PSP-P, and 4 had PSP-PGF. The authors found reduced cadence and increased cycle duration with a longer stance duration in PSP-RS compared to vPSP. With the dual task condition, they found an additional increase in stance phase in PSP-RS compared to the vPSP group. In addition to different methodologies for comparison groups, another important limitation of these studies is that straight walking on a gait mat may not elicit freezing episodes that are fully representative of real-world mobility impairments.

#### Balance in PSP-RS

Despite the fact that postural instability and falls are classic features of PSP, fewer studies have focused on static and dynamic balance compared to the number of studies of gait in PSP. Early studies focused on the contribution of vestibular dysfunction to balance impairment in PSP. In 2008 Liao et al. combined otolith-ocular reflexes (VORs) and vestibular-evoked myogenic potentials (VEMPs) while subjects with PSP-RS were seated on a dynamic chair capable of translations and rotation and found smaller translational VORs and smaller VEMPs in PSP compared to control subjects (16). The authors concluded that abnormal otolith reflexes may contribute to frequent falls in PSP. Using the sensory organization test (SOT), during which subjects stand in 6 conditions on a moveable force plate (Neurocom) platform (1. eyes open with stationary platform, 2. eyes closed with stationary platform, 3. eyes open with visual background movement, 4. eyes open with platform movement, 5. eyes closed with platform movement, and 6. eyes open with both background and platform movement), Ondo et al. showed that subject with PSP-RS performed worse than subjects with iPD on the total SOT score (17).They also found that subjects with PSP-RS had specific impairments in a pattern that they concluded suggested vestibular dysfunction (conditions 3, 4, 5, and 6 of the SOT). However, these are also the most challenging balance conditions in the SOT, nonspecific for vestibular loss.

Our group subsequently compared the sensory and motor responses of 12 subjects with PSP-RS, 12 postural instability and gait disturbance (PIGD)-matched subjects with iPD, and 12 healthy controls while sitting and standing on the same Neurocom moveable force plate platform system (13). We specifically examined subjects’ reactions to forward platform translations and toes-up platform tilts that resulted in backward sway. Compared to subjects with iPD, we found that subjects with PSP accurately perceived gravity when standing on a tilting surface, but could not accurately perceive toes-up platform tilts, and furthermore exerted less postural corrective motor responses in response to forward platform translations and toes up surface tilts. Taken together, we postulated that balance dysfunction in PSP is the result of abnormal central sensory integration, rather than a result of a primary vestibular deficit.

#### Combined gait and balance in mixed PSP phenotypes

More recently, Ali et al. combined gait and postural sway in a small number of PSP phenotypes versus age-matched controls using a 3D motion capture system (10). Sixteen patients with PSP (11 PSP-RS, 2 PSP-P, 2 PSP-SL, and 1 PSP-CBS) were compared with healthy controls using a 10-camera motion capture system and 41 body markers and ground-embedded force plates. They found a slower gait velocity, slower cadence, and longer double-support time in PSP that correlated with clinical disease severity on the PSP Rating Scale (PSPRS). They also noted larger antero-posterior sway, but there was no relationship between the clinical PSPRS scores and standing postural sway tasks. The findings suggest that static standing sway tasks may not fully capture dynamic balance impairments in PSP.

#### Data mining studies for classification of PSP versus PD

Machine learning approaches to classify gait in people with PSP from PD are the focus of two studies, one by Ricciardi (22) that uses data from a motion analysis system, and the other data from wearable Opal inertial sensors (APDM) by De Vos in 2020 (14). In the motion analysis study, straight walking data from 46 subjects with a mix of *de novo* PD, moderate PD, and unspecified PSP subtypes was compared. Freezing and turning data was excluded. In the initial machine learning classification attempt by Ricciardi, random forest and gradient boosted tree models correctly discriminated gait in those with PSP from iPD, with a sensitivity and specificity of 92.6 and 96.3 (random forest) and 96.3 and 92.6 (gradient boosted), respectively. However, because the disease duration differed largely between groups, the clinical utility of such classification is unclear. The subsequent machine learning study by deVos, 2020 used 6 wearable Opal sensors (placed on feet, wrists, sternum, and the lumbar region) to examine data from 4 PSP-RS subjects, 17 PSP-P subjects, 20 iPD subjects, and 30 healthy controls during a 2-min walk, sway on a firm surface with eyes closed, and a 3-m timed up and go task (14). The Opal triaxial sensors include accelerometers, gyroscopes, and a magnetometer. Subjects were tested on dopaminergic medication. The authors found that a random forest model with combined gait, sway, and timed up and go data predicted PSP versus PD with 86% sensitivity and 90% specificity. Sway, alone, did not discriminate the groups. This study was also limited by a variable disease duration in subjects. The average disease duration in the subjects with PD was 11.4 years, and only 2 years in the subjects with PSP. Additionally, machine learning approaches for classification of diseases can be of limited clinical utility when differences in the clinical features of the diseases under investigation are clinically apparent at baseline.

#### Radiological correlations with gait and balance measures in PSP

A 2016 study by Pasha et al. compared balance and radiological features in 17 PSP-RS and 12 PSP-P patients using a Biodex posturography system, which is a platform capable of tilting 20 degrees from the horizontal in all directions (19). They compared static limits of stability and dynamic stability in response to surface tilts with structural MRI features in PSP-RS and PSP-P and found that balance and radiological abnormalities were overall more severe in PSP-RS. This is consistent with evolution of the disease course, as we see that variants of PSP evolve with time and disease progression. In PSP-RS, they did not find any significant correlations between the PSPRS and specific areas of atrophy or between balance measures and imaging features. In PSP-P, the midbrain axial anterior–posterior diameter significantly correlated with the Tinetti Mobility Assessment total score and Gait subscore, but not with any dynamic posturography measures.

Palmisano et al. used a 3D motion capture system to examine anticipatory postural adjustments (APAs) for gait initiation in 26 subjects with PSP-RS and 14 age-matched controls and then correlates APA measures with metabolic activity on fluoro-D glucose (FGD) PET (18). Their study supported the findings of Amano and colleagues showing impaired APAs in people with PSP (11). Metabolic correlations were not significant after controlling for multiple comparisons, but the data suggested several trends toward significance such as an association between decreased regional caudate uptake and impaired APA control. The study was limited by a high rate of exclusion due to falls or total absence of the imbalance phase of the APA (8 out of 26 patients were excluded), and this highlights the major limitation of severity of disease in clinical trials in PSP.

A subsequent, multimodal imaging study in 19 subjects with PSP analyzed 3 T MRI markers of atrophy and white matter integrity on diffusion tensor imaging (DTI) and fortaucipir-PET metabolic imaging with principal components analysis (23). Various subtypes of PSP were represented including PSP-RS, PSP-P, PSP-SL, and PSP-CBS. Gait features of decreased stride length, increased step width, and longer double-support time related to DTI measures in the posterior thalamic radiation, external capsule, superior cerebellar peduncle, superior fronto-occipital fasciculus, body and splenium of the corpus callosum, and the sagittal striatum, to MRI volumes in frontal and precentral regions, and to flortaucipir-PET uptake in the precentral gyrus. Postural sway in standing, alone, did not correlate with imaging abnormalities, but this may be due to the mix of PSP phenotypes studied. In PSP-RS, alone, imaging and postural sway abnormalities did correlate. The authors note that a limitation of the study relates to the somewhat controversial use of flortaucipir PET in PSP, as it was optimized for the paired helical tau fragments in Alzheimer’s and is known to have off-target binding in PSP.

### Gait and balance as a biomarker of progression: longitudinal studies

Four longitudinal studies were included in this review (Table 2). These studies evaluated longitudinal changes in gait, balance, and cognition up to 1.5 years. Two studies observed that wearable sensors can serve as sensitive measures of PSP progression.

**Table 2:** Summary characteristics of the longitudinal included studies.

Study and Country	Participants	Subtypes of PSP and criteria for PSP	Cognitive and Mood status	Assessment	Walking aids	Follow-up	Clinical motor utcomes/other outcomes	Objective gait outcomes	Objective balance outcomes	Author’s conclusion
Abate et al. (26) Italy	PSP: *n =* 35, age (68.1 ± 5.4 years), disease duration (4.2 ± 2.5 years), men (*n =* 27), women (*n =* 8)	MDS PSP criteria. Richardson’s syndrome (*n =* 28), parkinsonism predominant (*n =* 5), freezing of gait predominant (*n =* 2)	Not reported	PSPRS, Wearable sensors (one on the back and one on each foot)	Ten (29%) participants required unilateral support to complete at least one of the required tasks.	Three-month follow-up for PSPRS and each objective variable	PSPRS total score did not show a significant change over the follow-up (0.78% increase), but significant differences were detected for the “emotional lability” item (36.54% decrease) and the “arising from chair” item (16.31% increase). PSPRS total showed moderate inverse correlations with gait speed (r = −0.434; *p* < 0.001), and with stride length, swing and turning velocity, 360° angle and 360° turning velocity and moderate correlations with gait double support, stance and turning duration. PSPRS gait/midline subscore presented a strong inverse correlation with gait turning velocity, moderate inverse correlations with gait speed, stride length, swing, 360° angle, 360° duration and 360° turning velocity and moderate direct correlations with gait double support time and stance.	The analysis from baseline to 3-month follow-up showed that cadence and cycle duration from the 2-min walking test presented a significant increase over time (by 3.69 and 3.94% respectively).	Not reported.	Results from the study demonstrated the change of objective gait parameters over a short-term follow-up. Wearable sensors can provide an objective, sensitive quantitative evaluation and immediate notification of gait changes in PSP.
Ghosh et al. (27) United Kingdom	PSP: *n =* 23, age (71.1 ± 8.6 years), disease duration (3 years), men (*n =* 14), women (*n =* 9) Healthy Control: *n =* 22, age (71.4 ± 7.6 years), disease duration (N/A)	PSPRS Richardson’s syndrome	ACE-R = 76.4 ± 10.9 FAB = 10.8 ± 3.9 VOS*P=*7.6 ± 3.2	PSPRS, UPDRS-III, Saccadometry	Not reported	1.2 years for PSPRS, UPDRS-III, Saccadometry and cognitive status	PSPRS showed a mean change over a year of 11.3 points (*p* < 0.001). UPDRS-III showed a mean change of 8.3 points (*p=*0.003) and mu (the inverse median latency for saccades) showed a mean decrease of 0.4 s^−1^(equivalent to an increase in latency of 0.02 s) (*p=*0.01). Cognition did not change significantly during the study period.	Not reported	Not reported	Patients show significant deterioration over one year using the PSPRS severity measure. Oculomotor function changed over one year, including the range of vertical gaze in the PSPRS.
Pereira et al. (28) United Kingdom	PSP: *n =* 28, age [69.2 (52–68) years], disease duration [1.9 (0.2–6.3) years], men (*n =* 15), women (*n =* 12) Healthy Control: *n =* 28, age [66.2 (56–72) years], disease duration (N/A)	MDS PSP criteria. Subtypes not reported	MoCA = 22.4 (12–30) MMSE = 26 (20–30) Fluency test (Semantic) = 21.8 (6–41) Fluency test (Phonemic) = 19.9 (6–50)	PSPRS UPDRS-III	Not reported	1.5 years for PSPRS and MDS-UPDRS-III and cognitive status. Assessment visits were done every 3 months.	The increase in MDS-UPDRS-III was statistically significant after 12 months (Δ = 11.75, SD = 12.31, *p* < 0.008) while the increase in PSPRS became significant 15 months after baseline assessment (Δ = 7.42, SD = 7.63, *p* < 0.008). The MoCA and MMSE scores did not show any enduring changes in scores over time.	Not reported	Not reported	Motor decline in PSP is consistently captured by clinical rating scales. These results support the inclusion of multiple follow-up time points in longitudinal studies in the early stages of PSP.
Sotirakis et al. (29) United Kingdom	PSP: *n =* 17, age [63 (51–73) years], disease duration [1.6 (0–6) years], men (*n =* 9), women (*n =* 8)	MDS PSP criteria. Subtypes not reported	MMSE = 26.2 (20–30)	Kinematic gait and posture features collected by a body-worn IMU	Not reported.	1 year, over five visits at 3-month intervals.	Not reported.	There was a significant change in mean turn velocity, SD of Stride length and mean toe off angle. These three features served exclusively as predictors of progression on a mathematical model used to predict MDS-UPDRS-III and PSPRS-motor. Strongly significant differences from baseline were apparent 3 months earlier in these models than in the actual scores	Not reported.	Data from wearable IMU arrays coupled with mathematical modeling can be used to track progression of PSP, complementing established clinical rating scales. In this study, the reduced variability in the modeled data allowed a progression signal to be discerned 3 months earlier than would otherwise be expected.

ACE-R = Addenbrooke’s Cognitive Examination—revised; FAB = Frontal Assessment Battery; MDS-UPDRS-III = Movement Disorders Society Unified Parkinson’s Disease Rating Scale part III motor subscale score; MoCA = Montreal Cognitive Assessment; MMSE = Mini-mental State Examination; NINDS-SPSP: National Institute of Neurological Disorders and Society for Progressive Supranuclear Palsy criteria for PSP; PSPRS = PSP rating scale; UPDRS-III = Unified Parkinson’s Disease Rating Scale part III motor subscale score; VOS*P=*Visual Object and Space Perception Battery.

An early study by Ghosh et al. (27) previously examined PSPRS and oculomotor function changes in 23 subjects with PSP Richardson syndrome over 14 months. They found significant changes on both the PSPRS and vertical eye movements *via* saccadometry during that period, but objective gait and balance outcomes were not used.

As part of a larger, longitudinal study in the United Kingdom, the “OxQuip” study, Pereira et al. examined longitudinal changes in motor and cognitive symptoms on clinical scales in PSP (28), and then Sotirakis et al. (29) built upon this background with longitudinal monitoring of PSP with 6 body-worn, inertial measurement units (IMU) sensors (“Opals,” by APDM). Pereira analyzed the PSPRS, MDS-UPDRS 3, MOCA, and MMSE in 28 subjects with possible or probable PSP by 2017 MDS criteria (with symptom onset at an average of 1.9 years prior to enrollment, but PSP subtypes were not specified) at visits every 3 months for 18 months. The gait and midline sub-score of the PSPRS was the earliest score to change and this change was observed at *6 months*. This study experienced a drop-out rate of approximately 50% due to progression of illness, death, or change in diagnosis (the latter in only one subject). Other limitations of this study were the lack of pathological diagnoses and the fact that dopaminergic medication use was not accounted for at the time of assessments.

Sotirakis et al. then applied 6 wearable IMU Opal sensors to the wrists, feet, sternum, and lumbar region for longitudinal measurement in 27 subjects with PSP of the PSP-RS and PSP-P subtypes. The Opal sensors were applied for a 2-min walk with 180 degree turns and for a postural sway task for 30 s with eyes closed. Data from 17 participants was sufficient for analysis of visits at 3-month intervals for 12 months. Linear regression revealed that a model incorporating turn velocity, stride length standard deviation, and toe off angle detected statistically significant progression at visit 4, which was *3 months earlier* than the clinical PSP Rating Scale, alone. This was an important first study to quantify disease progression in PSP using wearable sensors. An important limitation of this study is the lack of accounting for the potential influence of physical therapy interventions on progression.

A subsequent study by Abate et al. (26) also examined disease progression in PSP using Opal inertial sensors, and correlated kinematic data to the PSPRS. Twenty-three subjects were assessed for progression, and PSP phenotypes included in this study were PSP-RS (80%), PSP-P (14%), and PSP-PGF (6%). In this study Opals were applied to the feet and lumbar area only. At the 3-month follow-up, cadence and gait cycle duration from a two-minute walking task worsened significantly, although the total PSPRS did not worsen significantly, except for the specific “arising from chair” sub-item that did worsen significantly. A strength of this study is the use of fewer sensors, which improve ease of clinical use. An important limitation of this study is that only 29% of the subjects with PSP needed unilateral assistance for gait (i.e., a cane or a helper holding onto one limb), so the population only encompassed relatively mild disease presentations of PSP.

The Sotrirakis and Abate studies (26, 29) both suggest that wearable sensors may be important and more sensitive detectors of disease progression than the PSPRS. Both studies also found that dynamic gait parameters, rather than balance parameters, are related to disease progression. The authors hypothesize that dynamic instability outweighs static instability for assessment of progression, at least in the relatively early stages of the disease. It is important to acknowledge that more wearable sensor assessment of static balance is needed to better understand progression, particularly in more advanced stages of PSP.

### Rehabilitation intervention studies

Fourteen rehabilitation intervention studies were included in this review (Table 3). These studies evaluated the effect of different interventions (balance training, home-gait exercise body-weight supported treadmill gait, virtual reality intervention, Robot-assisted walking, and cerebellar rTMS) on gait and balance outcomes in people with PSP. No study used wearable sensors to evaluate gait and balance impairments.

**Table 3:** Summary characteristics of the rehabilitation interventions included studies.

Study and Country	Participants	Experimental	Control	Measurement tools	Walking aids	Clinical motor outcomes/other outcomes	Objective gait outcomes	Objective balance outcomes	Adverse events	Author’s conclusion
Clerici et al. (30) Italy	*n =* 24 PSP patients *Experimental group:* age 69.9 ± 5.2, disease duration 4.1 ± 1.4 years *Control group:* age 72.5 ± 6.1; disease duration 4.0 ± 1.2 years	Lokomat^®^ Training (20 min), maximum velocity tolerated, not exceeding 2.5 km/h, 5 times/week for 4 weeks.	Treadmill Training with visual and auditory cues (20 min), maximum velocity tolerated, not exceeding 2.5 km/h 5 times/week for 4 weeks.	PSPRS, BBS, 6MWT and number of falls	Not reported	Total PSPRS, PSPRS-gait, BBS, 6MWT and number of falls improved significantly by the end of the training programs in both groups. PSPRS-limb score improved significantly only in control group.	Not reported	Not reported	Not reported	Aerobic, motor-cognitive and goal-based rehabilitation treatments based on a multidisciplinary and intensive approach are useful for PSP patients, even without the support of expensive robotic technologies such as Lokomat®
Croarkin et al. (31) United States	*n =* 1 atypical PSP patient Age 63 years old, disease duration 11 years	Boxing, stepping tasks and treadmill training, 20 min for each 2 times/week, for 6 weeks	No	Computerized posturography, 10 camera DX system (Vicon Motion Systems), performance-based tests of timed stepping and unilateral squats	Not reported	Gains in strength were noted by improvements in his home exercise regimen.	Not reported.	Foot clearance scores increased around 0.2 to 2 cm bilaterally. Results on the repeated stepping test and the squats during unilateral stance also improved. Increased speed, symmetry, and accuracy were recorded.	Not reported	The intervention improved balance, eye-body coordination and strength in a high functioning patient with PSP.
Dale et al. (32) United States	*n =* 2 PSP patients Age and disease duration not reported	10 days of active Cerebellar rTMS (4,000 pulses were delivered with a 70 mm figure-of-8 coil at 10 Hz, 4 s on, 8 s off, 100 trains, machine output 90e110% of RMT, pending tolerability) plus 10 days of Sham treatment	No	Cerebellar brain inhibition (CBI) assessment, posturography.	Not reported	CBI increased by 50% in subject 1 and by 32% in subject 2.	Not reported	Subjects’ backward stability improved when standing on a force plate, as evidenced by reduction of the backward center of pressure excursion (less sway in the posterior direction).	Not reported	Cerebellar rTMS with neuronavigation may result in improved postural stability in PSP.
Di Pancrazio et al. (33) Italy	*n =* 10 PSP patients Age 69 ± 7 years, disease duration not reported	20–30% body-weight supported treadmill gait training (20 min) plus mechanical acoustic vibrations 3 times/week, for 8 weeks	No	PSPRS, BBS, Baropodometry static and dynamic, Stabilometry	Not reported	PSPRS showed improvement of the motor score posture item (*p=*0.01), and in the motor score postural stability item (*p=*0.01). BBS score varied from a 37.7 ± 12.1 at the baseline to a score of 47,6 ± 9.2 at the end of treatment (*p=*0.02).	Not reported	Stabilometry test showed a significant improvement of the distribution of the load in percentage.	Not reported.	The rehabilitation program was efficient on posture and on walking quality. The patients showed an increase in walking speed, greater stability and a consequent reduction in the risk of falling.
Irons et al. (34) United States	*n =* 1 PSP patient Age 67 years old, disease duration 1.5 years	17–21% body-weight supported motor-assisted elliptical training, time and speed progressively increased to 30 min and 50 rpm. 3 times/week, for 8 weeks	No	6MWT, FOG-Q, SSC (self-selected comfortable treadmill speed (m/min)) Oxygen cost of SSC walk speed	Not reported	Improvement of 82.9 m from pretraining on the 6MWT distance. The oxygen cost of SSC gait speed improved 6.8% between pretraining (0.44 mL·kg^−1^·m^−1^) and post training (0.41 mL kg^−1^ m^−1^). This improvement in oxygen cost was sustained 1 month later (0.41 mL·kg^−1^·m^−1^).	Not reported	Not reported	Not reported.	The intervention resulted in improved gait efficiency (oxygen cost of SSC gait speed) and distance traversed (6MWT).
Matsuda et al. (35) Japan	*n =* 20 PSP patients Age 72.3 ± 6.2 years, disease duration 2.4 ± 1.5 years	Balance training, resistance training, range of motion (ROM) exercises, stretching, walking exercises, and ADL training, 60–80 min/day 5 times/week, for 4 weeks	No	PSPRS, BBS, TUG, Pull test, comfortable and maximum gait speed	Not reported	Improvements of PSPRS gait and midline total scores (*p=*0.004, r = 0.645), were found after intervention. BBS showed significant improvements in the items of reaching forward with outstretched arm (*p=*0.011, r = 0.566), turning to look behind (*p=*0.039, r = 0.461), turning 360 degrees (*p=*0.046, r = 0.447), standing with one foot in front (*p=*0.047, r = 0.445), and standing on one foot (*p=*0.009, r = 0.588).	No statistically significant difference was found for comfortable and maximum gait speed.	Not reported.	Not reported	A multiple therapeutic exercise program can improve the balance function in patients with PSP.
Nicolai et al. (36) Germany	*n =* 8 PSP patients Age 66.4 ± 6.2 years, disease duration 6.2 ± 4 years	Balance exercises (sitting, standing, stepping) plus auditory cues (45 min) 3 times/week, for 6 weeks	No	BBS, TUG, 5CR, UPDRS-III	Five participants used a walking aid, and two participants were wheelchair-bound but able to stand upright without another person’s help.	Median values of the BBS improved by 25.7% (*p=*0.016) from pre to post intervention.	Not reported	Not reported	No adverse events.	The intervention is both feasible and associated with functional and psychosocial improvements for PSP patients.
Pilotto et al. (37) Italy	*n =* 20 PSP patients Age 67.8 ± 11.7 years, disease duration 3.6 ± 1.8 years	Each patient received both rTMS and sham cerebellar single session stimulations in randomized order in two different sessions performed at the same time of the day, separated by at least 2 weeks.	No	Tinetti test, SPPB, TUG, FRT, IMUs	No	Not reported	Not reported	No differences in baseline performances in instrumented tests were detected for each task between real and sham stimulation. In both eyes closed conditions, the participants were able to stay longer without support after the real rTMS, compared to sham stimulation	Not reported	Results suggests a beneficial effect of a single session of cerebellar rTMS stimulation on measures of postural instability in PSP patients
Sale et al. (38) Italy	*n =* 5 PSP patients Age 74 ± 4 years, disease duration 3.8 ± 1.2 years	Robot-assisted walking (45 min) 5 times/week, for 4 weeks	No	3D-Gait analysis	Not reported	Not reported	Gait velocity and cadence improved, respectively, by 15 and 23.8%. It was also shown an improvement of 11% in step length left and of 35% in step length right, besides a decrease Of 9% of Step width. Due to a small sample size, no statistical significance was found in all the analyzed parameters.	Not reported	No adverse events.	The positive results on improvement in spatiotemporal parameter of the PSP subject by the Robot Therapy, the lack of side effects strongly recommends extending the use of a Robot Therapy in the recovery of gait performance.
Seamon et al. (39) United States	*n =* 1 PSP patient Age 65 years old, disease duration 5 years	Xbox Kinect virtual exergaming (60 min) 2 times/week, for 6 weeks	No	BBS, FFABQ, FGA, 10 Meter walk test, TUG	Not reported		Fastest comfortable gait speed changed from 1.16 m/s to 1.05 m/s on the 10 Meter Walk Test. Balance function remained stable and no declines below fall risk cut offs listed for elderly individuals.	Not reported	Not reported	Results of the study demonstrates the feasibility of an intervention using a virtual gaming system to help maintain functional mobility, balance and independence for an individual with PSP.
Shima et al. (40) Japan	*n =* 1 PSP patient Age 70 years old, disease duration 1.6 years	tACS plus 4-min self-paced backward gait on the treadmill. A combination of sham stimulation (Intervention A), gait-synchronized cerebellar tACS (Intervention B), and cerebellar tACS asynchronized with gait using the inverted phase as a control condition (Intervention C) was performed. The order of the interventions was A–C, with an interval of more than a week between the interventions. 10 times backward training, 2 times/week, for 5 weeks	No	PSPRS, TUG, mini-BESTest, FES, modified FES ABC scale, VAS, CBI	No	The short-term intervention elucidated that Intervention B improved the time of TUG and the total score of the mini-BESTest, whereas interventions A and C did not. The VAS revealed the largest improvement in general motor symptoms in Intervention B and improvements in gait and balance functions evaluated using TUG and mini-BESTest. PSPRS was also improved, especially in the subscale of the “Gait and midline,” along with the VAS improvement for general symptoms The FES and modified FES scores increased after long-term intervention. The ABC scale also showed an increase in scores: 490 at pre-intervention and 640 at post-intervention. CBI using paired TMS of the left M1 and right cerebellum was improved at inter-stimulus interval of 3,5, and 10 ms, suggesting that the function of the right cerebellum was recovered	Not reported	Not reported	No	The results demonstrates that backward gait training combined with synchronized cerebellar tACS can be a promising treatment for improving the motor symptoms of PSP
Suteerawattananon et al. (41) United States	*n =* 1 PSP patient Age 62 years old, disease duration >5 years	15% body-weight supported treadmill gait training (90 min) 3 times/week, for 8 weeks	No	BBS, TUG, 15.2-m (50-ft) walk test, FRT, LOS, spatiotemporal gait measures	Sometimes the patient carried a cane for better balance.	The BBS score increased from 45 at the beginning of the training to 49 at midpoint, but it decreased to 47 by the end of the program.	Gait speed increased from 73.40 ± 10.47 cm/s to 100.05 ± 0.78 cm/s after training. Step length of the left and right legs improved from 43.76 ± 5.52 cm and 49.66 ± 4.32 cm to 51.27 ± 0.44 cm and 58.74 ± 3.80 cm, respectively.	Static balance in reaching forward increased 3.63 cm.	Not reported	The intervention might be an appropriate apparatus to reduce falls and improve balance and mobility in patients with PSP.
Wittwer et al. (42) Australia	*n =* 5 PSP patients Age ranging from 54–74 years old, disease duration ranging from 1.1–12.8 years	Home-based gait and Step training plus music and auditory cues (60 min) 2 times/week, for 4 weeks	No	Spatiotemporal gait measures (GAITRite)	2 patients occasionally used walking aid (single point stick, 4 wheeled frame)	Not reported	Gait velocity and stride time improved for three patients. Stride time variability improved for four patients. Clinical significance was found for gait velocity in one patient.	Not reported.	No	The intervention was feasible for people living with mild to moderately severe PSP and was associated with improvements including reduced variability in temporal and spatial measures of walking.
Zampieri et al. (43) United States	*n =* 19 PSP patients *Experimental group:* age 71.2 ± 5.2, disease duration 3.4 ± 2.6 years *Control group:* age 67.5 ± 7.2; disease duration 4.4 ± 2.8 years	Balance exercises plus eye movement and visual awareness training (60 min) 3 times/week, for 4 weeks	Balance exercises only (60 min) 3 times/week, for 4 weeks	Kinematic gait measures through the 2.4 m (8 ft) walk test, TUG	Not reported	TUG score decreased more in the experimental group than in the control group. No statistically significant differences between the groups were observed.	A significant decrease in stance time was observed in the experimental group. There was a significant improvement in walking speed on the 8-ft walk test in the treatment group but not in the control group.	Not reported.	Not reported	Balance exercises coupled with eye movement exercises may improve gait in people with PSP. Improvements in spatial gait parameters, gait speed, and TUG scores were observed for participants who received balance and eye training.

ABC scale = Activities-Specific Balance Confidence scale; BBS = Berg Balance Scale; CBI = Cerebellar brain inhibition; FES = falls efficacy scale; FFABQ = fear of falling avoidance belief questionnaire; 5CR = Five chair rise test; FOG-Q = Freezing of Gait Questionnaire; FGA = Functional gait analysis; FRT = Functional reach test; LOS = limits-of-stability test; PSPRS = PSP rating scale; SPPB = Short Physical Performance Battery; 6MWT = 6 Minutes Walking Test; tACS = transcranial alternating current stimulation; TUG = Timed-up and Go test; UPDRS-III = Unified Parkinson’s Disease Rating Scale-motor subscale, VAS = visual analog scale.

#### Effect of rehabilitation interventions on spatiotemporal gait metrics in PSP

Changes in spatiotemporal gait metrics were observed in six rehabilitation studies (31, 38, 39, 41–43). Of these studies, only one had a sample size of 19 people with PSP (43), the other studies were case report that investigated the effects of treadmill training and boxing (31), robot-assisted walking (38), virtual reality (39), treadmill training with body weight support (41), and cueing step-training (42) on spatiotemporal gait parameters such as gait speed, stride length, and cadence. Although these studies have shown changes in gait speed, stride length, and cadence after a short period of intervention, ranging from 8 (42) to 24 sessions (41), it is important to emphasize that the data from these case studies do not allow causal conclusions on the effects of these mode of rehabilitation in PSP. Therefore, caution should be exercised when interpreting these findings as they cannot be generalized to the entire PSP population. Thus, robust clinical trials are needed to investigate the effects of rehabilitation intervention on spatiotemporal gait parameters in people with PSP.

Zampieri et al. (43) assessed the effect of a rehabilitation intervention in PSP on kinematic gait parameters (stance time, swing time, and step length) by tracking foot motion using electromagnetic sensors. Nineteen people moderately affected by the PSP were assigned to either a treatment group (balance plus eye movement exercises, *n =* 10) or a comparison group (balance exercises only, *n =* 9). Although the authors did not find a difference between groups for any gait parameter, the within-group analysis revealed significant improvements in stance time and walking speed for the treatment group, whereas the comparison group showed improvements in step length only. These preliminary findings support the use of eye movement exercises as a complementary therapy for balance training in the rehabilitation of some gait parameters in people with PSP; however, future clinical trials powered at a higher level are needed to confirm these results.

#### Effect of rehabilitation interventions on clinical balance in PSP

Changes in clinical balance were observed in 6 rehabilitation studies (30, 32, 33, 35, 36, 40). These studies had a sample size ranging from 1 (40) to 24 (30). The number of sessions ranged from 10 (40) to 24 sessions (33). Most studies used the Berg Balance Scale (BBS) to assess clinical balance, while one study used the Mini-BESTest (40). Different interventions were used such as treadmill training (30), body-weight supported treadmill gait training (33), balance and resistance training (35), cueing balance-exercises (36), and backward gait training combined with gait-synchronized transcranial alternating current stimulation (tACS) (40).

Clerici et al. (30) observed that 20 sessions of treadmill training with visual cues and auditory feedback, both with (*n =* 12) and without (*n =* 12) the use of a robotic device, significantly improved the BBS scores in people with PSP. The authors concluded that both interventions have similar effects on clinical balance of this population, thus, the usefulness of an aerobic, sensory-feedback approach for the rehabilitation of patients suffering from PSP may be implemented in future clinical trials. Di Pancrazio et al. (33) tested the effect of 24 sessions of a rehabilitative program combining sensorimotor exercises (postural control, vibration, and cues) on postural instability of ten people with PSP. The authors observed that the combined rehabilitative program produced improvement in the BBS score and this clinical balance improvement persisted also in the follow-up phase after 30 days. Although the authors suggest that this specific rehabilitation program could improve postural instability in people with PSP due to intensive sensory stimulation involved in the intervention protocol, the failure to use a control group can make it impossible to draw meaningful conclusion from this study. Likewise, Matsuda et al. (35) applied 20 sessions of balance and resistance training in 20 people with PSP without the use of a control group. They also observed beneficial effects on the BBS score.

Although we do not know exactly the positive effects of progressive resistance strength training on people with PSP, there is strong evidence of benefit of this intervention in people with PD (44, 45). Two years of progressive resistance strength training were more effective than 2 years of non-progressive exercise in decreasing the motor symptoms of patients with mild-to-moderate PD (44). Our previous studies have demonstrated that combining balance exercises with progressive resistance strength training is more effective than progressive resistance strength training alone in decreasing motor symptoms of PD (46), as well improving clinical balance on the Balance Evaluation Systems Test (BESTest), mobility (timed-up-and-go test), and fear of falling in people with mild-to-moderate PD (47). Thus, a combined balance and progressive resistance training intervention would be more effective for people with PSP than progressive resistance training alone. Future controlled and randomized studies should test this intervention in PSP.

Nicolai et al. (36) tested the effects of 18 sessions of audio-biofeedback training on the BBS score in 8 people with PSP. This study used a new device that was well accepted for the participants and no adverse events occurred. Although the authors observed a significant improvement in the BBS score, which remained significant at the 4-week follow-up, the lack of a control group makes it difficult to be certain that the improvement in the BBS scores was caused by the audio-biofeedback training and not by other variables in the intervention. Thus, future powered and robust clinical trials are necessary to investigate the effects of sensory-feedback rehabilitation intervention on the clinical and objective balance of people with PSP.

Only one study investigated the effects of rehabilitation on freezing of gait (FOG) in people with PSP (34). We know FOG negatively impacts balance and functional gait in this population (48, 49). Irons et al. (34) observed that 24 sessions of a motor-assisted elliptical trainer with body weight support decreased FOG in a 67-year-old man with PSP. However, 1 month without training revealed worsening of his FOG, although the improved oxygen cost during training was sustained at 1-month follow-up. This case study is the first to document FOG improvement after a motor-assisted, elliptical training program for an individual with PSP, and future studies with a larger sample size are needed to investigate the possible benefits of this structured rehabilitation for people with PSP.

Interventions that involve cognitive and balance exercises should be applied to decrease FOG in people with PSP. People with PSP have more fear of falling, cognitive and balance impairments, and falls compared to people with PD (50). Our previous studies have demonstrated that 36 sessions (47, 51) or 18 sessions (52, 53) of challenging motor-cognitive balance training improved spatiotemporal gait parameters, anticipatory postural adjustments, postural stability, as well decreased FOG severity (51) and improved cognitive function in people with mild-to-severe PD. These interventions are challenging and need to be applied individually. As people with PSP are at higher risk of falling compared to those without PSP (49, 50), these exercises would be performed individually and with a body-weight support system (e.g., ZeroG) (54), in an attempt to significantly improve gait, cognition, FOG, and balance in a safe way. Thus, future, randomized, clinical trials are encouraged to implement this motor-cognitive rehabilitation strategy.

Using a different intervention approach, Shima et al. (40) assessed the effect of 10 sessions of rehabilitation (backward gait training) combined with gait-synchronized, cerebellar transcranial alternating current stimulation (tACS) on the MiniBESTest score in a 70-year-old woman with PSP-Richardson’s syndrome. Initially, the participant underwent short-term intervention with combined training of backward gait with synchronized cerebellar tACS, asynchronized, or sham stimulation according to the N-of-1 study design. Synchronized tACS training demonstrated an improvement in the MiniBESTest scores, whereas asynchronized or sham stimulation did not. The additional long-term interventions of combined backward gait training with synchronized cerebellar tACS demonstrated further improvement in MiniBESTest. Although this case study results can be difficult to replicate due to the sample size, it describes a novel approach for clinical balance in a patient with PSP-Richardson’s syndrome, as backward gait training with synchronized cerebellar tACS may be a promising therapeutic approach due to pathophysiology of disease involving cerebellar dysfunction (55, 56) and backward falls. Robust, prospective clinical trials are needed to test this new approach in people with PSP.

Although clinical balance was assessed in 6 rehabilitation studies (30, 33–36, 40), 3 of these studies used a quasi-experimental design (33, 35, 36), 2 used a case report (34, 40, 44–47), and only one study used an experimental design (30). Thus, the effects of balance- and gait-focused rehabilitation for people with PSP are still unknown due to small sample sizes. Future powered, prospective and robust clinical trials are needed to investigate the effects of rehabilitation interventions on clinical balance of the people with PSP.

#### Effect of rehabilitation interventions on objective balance measures in PSP

The effect of rehabilitation interventions on balance posturography has been investigated only in 2 studies, both of which used cerebellar transcranial magnetic stimulation (TMS) in people with PSP (32, 37). Neuroimaging and neuropathology studies have revealed a reduced volume of the cerebellum with tau accumulation (55, 56) that may be responsible for impaired balance and gait in patients with PSP. Thus, stimulatory cerebellar TMS may be a promising tool to improve balance and motor control in people with PSP.

Dale et al. showed that 10 sessions of cerebellar repetitive TMS (rTMS) improved backward postural stability when 2 subjects with PSP-RS stood on a force plate (Neurocom), as evidenced by reduction of the backward center of pressure excursion (less sway in the posterior direction) (32). The authors also observed that the 10 sessions of cerebellar rTMS increased cerebellar-brain inhibition by 50% in subject 1 and by 32% in subject 2. The rTMS protocol was well tolerated. Cerebellar rTMS may improve postural stability, but larger future studies are needed. One such study is currently enrolling (NCT04468932).

A recent study of cerebellar TMS compared theta burst TMS with sham cerebellar, single session stimulation in a randomized order in 2 different sessions in 20 people with PSP (37). Before and after stimulation, static balance was evaluated with instrumented (lower back accelerometer) 30-s trials in semi-tandem and tandem positions. In tandem and semi-tandem tasks, active stimulation was associated with increase in time without falls. In addition, postural sway area, velocity, acceleration, and jerkiness was improved only after theta burst TMS, compared to sham stimulation. These preliminary data suggest that cerebellar theta burst TMS has significant effect on postural stability in people with PSP, when assessed with mobile digital technology. The authors suggest that these results should motivate larger and longer trials using non-invasive brain stimulation for people with PSP. Future powered, prospective and robust clinical trials are needed to investigate the effects of cerebellar TMS on outcomes of this population.

## Conclusion

Objective measurement to quantify gait and balance and effects of rehabilitation on gait and balance in PSP is a rapidly growing field, with potential uses to classify of early parkinsonism, monitoring progression, and documenting effects of rehabilitation. A natural tension exists between lab-based, comprehensive 3D motion capture of gait and force plate measures of postural sway and wearable inertial sensors (57). The former yields laboratory, gold-standard data, but is impractical for clinical trials. Wearable sensors are currently being used in clinical trials of balance and gait and have the potential for home-based daily life monitoring of mobility (7–9).

In this narrative review we examined: (a) cross-sectional studies in PSP focused on quantifying step initiation and steady state gait and postural sway for standing balance that relate to disease progression and imaging features, and (b) the use of objective gait and balance metrics as endpoints for rehabilitation and brain stimulation intervention studies in PSP. This review suggests several potential practical applications: for example, abnormal anticipatory postural adjustments when initiating gait suggest medio-lateral tasks should be a focus in rehabilitation for PSP, not just backward postural instability, and body-worn sensors for longitudinal monitoring may detect relevant gait changes 3 months earlier than the PSP Rating Scale.

However, studies of objective measurement of gait and balance in PSP suffer from several limitations common to studies of rare diseases: small sample sizes, no pathological confirmation of diagnoses, lack of multi-center studies, lack of replication, lack of long-term follow-up, and unclear subtyping of PSP classification. A particular note of caution when interpreting PSP classification studies: it is important to consider what particular mix of PSP subtypes is being evaluated and if it is reasonable to lump such variants together for analysis. PSP-RS, PSP-P, and PSP-PGF are the most represented variants, and these variants can look rather different clinically. Little is known about any subtle gait or balance abnormalities that objective metrics may elucidate in other categories of PSP, such as possible PSP speech and language, possible PSP with predominant corticobasal syndrome, and other categories that are suggestive of early PSP. It is also important not to consider variant subtypes of PSP as static categories, but rather milestones along a progression to eventual development of probable PSP-RS.

Regarding rehabilitation interventions, due to small sample sizes, low statistical power and comparatively low methodological rigor (lack of a control group and case series) in the studies included in this review, the effectiveness of rehabilitation interventions on objective measures of gait and balance and clinical balance still needs to be confirmed. Although we still do not know the optimal content of exercise (dosage, frequency, intensity, time, and type) for people with PSP, the most of the studies included in this review have used gait training, balance training, and sensory feedback training of gait and balance. Thus, there is a need to understand if rehabilitation interventions may have positive impact in a large population with PSP in future randomized clinical trials.

Looking forward, longitudinal monitoring with objective gait and balance metrics from body-worn sensors should be incorporated into future clinical trials with PSP, complementing the PSPRS clinical scale that has traditionally been used as the primary endpoint. Studies of correlations between objective measures of gait and balance and imaging features in PSP are in early stages, but are likely to grow in the coming years. Exercise regimens in PSP are often modified from PD or stroke regimens, and development of rehabilitation targeted specifically to the balance and gait impairments in people with PSP are needed. Exercise and other intervention studies benefit from objective gait and balance endpoints, but need replication with multisite application and long-term follow-up.

## Author contributions

MD conceived and designed the study. FA and CS-B collected and organized the data. MD and CS-B drafted the manuscript. FH critically revised the manuscript. All authors contributed to the article and approved the submitted version.

## Funding

National Institute of Neurological Disorders and Stroke; 1K23NS121402-01A1 (MD).

## Conflict of interest

FH is a part-time employee of Clario, that makes APDM Opals for clinical trials. This potential conflict has been managed by Oregon Health and Science University.

The remaining authors declare that the research was conducted in the absence of any commercial or financial relationships that could be construed as a potential conflict of interest.

## Publisher’s note

All claims expressed in this article are solely those of the authors and do not necessarily represent those of their affiliated organizations, or those of the publisher, the editors and the reviewers. Any product that may be evaluated in this article, or claim that may be made by its manufacturer, is not guaranteed or endorsed by the publisher.
